# *SGMS2* in primary osteoporosis with facial nerve palsy

**DOI:** 10.3389/fendo.2023.1224318

**Published:** 2023-10-11

**Authors:** Sandra Pihlström, Sampo Richardt, Kirsi Määttä, Minna Pekkinen, Vesa M. Olkkonen, Outi Mäkitie, Riikka E. Mäkitie

**Affiliations:** ^1^ Folkhälsan Institute of Genetics, Helsinki, Finland; ^2^ Research Program for Clinical and Molecular Metabolism, Faculty of Medicine, University of Helsinki, Helsinki, Finland; ^3^ Children´s Hospital, University of Helsinki and Helsinki University Hospital, Helsinki, Finland; ^4^ Minerva Foundation Institute for Medical Research, Helsinki, Finland; ^5^ Department of Anatomy, Faculty of Medicine, University of Helsinki, Helsinki, Finland; ^6^ Department of Molecular Medicine and Surgery and Center for Molecular Medicine, Karolinska Institutet, Stockholm, Sweden; ^7^ Department of Otorhinolaryngology – Head and Neck Surgery, Helsinki University Hospital and University of Helsinki, Helsinki, Finland

**Keywords:** SMS2, SGMS2-related osteoporosis, sphingomyelin metabolism, bone and neural tissue, sphingolipids

## Abstract

Pathogenic heterozygous variants in *SGMS2* cause a rare monogenic form of osteoporosis known as calvarial doughnut lesions with bone fragility (CDL). The clinical presentations of *SGMS2*-related bone pathology range from childhood-onset osteoporosis with low bone mineral density and sclerotic doughnut-shaped lesions in the skull to a severe spondylometaphyseal dysplasia with neonatal fractures, long-bone deformities, and short stature. In addition, neurological manifestations occur in some patients. *SGMS2* encodes sphingomyelin synthase 2 (SMS2), an enzyme involved in the production of sphingomyelin (SM). This review describes the biochemical structure of SM, SM metabolism, and their molecular actions in skeletal and neural tissue. We postulate how disrupted SM gradient can influence bone formation and how animal models may facilitate a better understanding of *SGMS2*-related osteoporosis.

## Introduction

Osteoporosis is a chronic bone disease with a significant global impact on morbidity and mortality. The defining characteristics are low bone mineral density (BMD) and disturbed bone microarchitecture, which enhance the risk of fragility fractures (1). Most often, polygenetic factors rather than single gene abnormalities are thought to influence a person’s bone health and risk of osteoporosis (2). Nevertheless, several uncommon monogenic types of osteoporosis have been found (1–3).

One of the most recently identified genes to cause a rare monogenic form of osteoporosis is *SGMS2*, which codes for the enzyme sphingomyelin synthase 2 (SMS2) (4). SMS2 catalyzes the production of sphingomyelin (SM), a type of sphingolipid that serves as a major component of the cell and Golgi membranes. Heterozygous mutations in the *SGMS2* gene (p.Arg50*, p.Ile62Ser, p.Met64Arg) cause a rare skeletal disorder termed calvarial doughnut lesions with bone fragility (CDL) with or without spondylometaphyseal dysplasia, with low BMD, neonatal fractures, long-bone deformities, and short stature (OMIM #126550). In addition to the skeletal manifestations, several patients experience neurological symptoms, the most frequent being transitory, spontaneously resolving, and recurrent cranial nerve palsies (4–8). The clinical presentation and disease severity is highly variable and dependent on the underlying *SGMS2* variant. Therefore, it is likely that the *SGMS2* variants could be causal in further primary osteoporosis patients with yet an unidentified genetic cause and the range of phenotypic manifestations significantly greater than has been previously described. Hence, it is of great importance to further understand how SM metabolism and lipid distribution affect bone development and metabolism. In this review, we aim to provide a comprehensive overview of the research topic and bring the latest knowledge of SMS2 and SM metabolism in skeletal and neural tissue to clinicians and researchers working in skeletal and neurological research fields. In addition, we underline the importance of developing *sgms2* modified animal models for studying molecular and cellular mechanisms underlying *SGMS2*-related bone fragility with neurological features.

## Sphingomyelin, a type of sphingolipid

Sphingolipids are fundamental structural components in cell membranes, including the plasma membrane, Golgi apparatus and endosome membrane. Sphingolipids contribute to the characteristic key properties of these membranes including the protective barrier function of the plasma membrane (9). Sphingolipids are essential in cell signaling, by both forming lipid rafts that play a crucial role in protein sorting and receptor‐mediated signal transduction and by serving as stores for signaling molecules. Sphingolipid metabolites are for instance important mediators in the signaling cascades involved in differentiation, apoptosis, proliferation, inflammation, and senescence (10).

**Figure 1:**
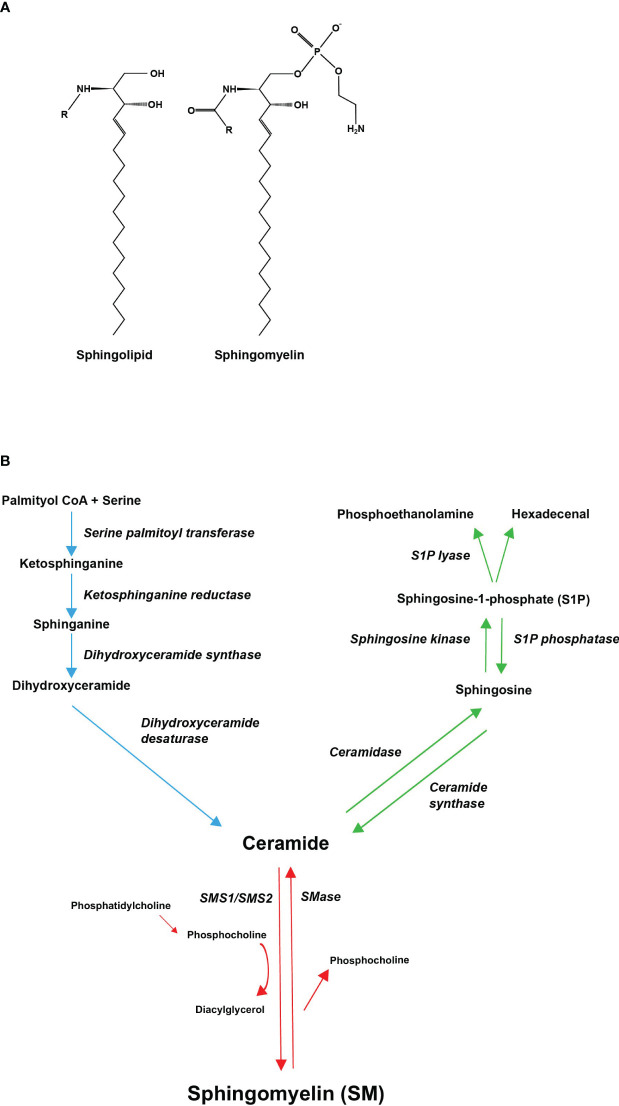
Molecular structures of sphingolipids and the metabolic pathway of sphingomyelin. **(A)** Every sphingolipid has a single sphingosine backbone, which is an 18-carbon alkyl chain with one to three hydroxyl groups and one amino group. Different functional groups (R) can bind to the amino group. Sphingomyelin is composed of the sphingosine backbone bound to a fatty acid tail and a phosphocholine group via its amino group and terminal hydroxyl group, respectively. **(B)** Sphingomyelin can be synthesized *de novo*, through ceramide, from saturated fatty acids (palmitic acid) (demonstrated with blue arrows). Ceramide serves as a substrate for sphingomyelin synthase (SMS), which catalyzes the transfer of phosphocholine, cleaved from phosphatidylcholine (PC), onto ceramide generating SM and diacylglycerol (DAG) (demonstrated with red arrows). Ceramide can also be converted to sphingosine-1-phosphate (S1P) through hydrolysis of its fatty acid residue and subsequent phosphorylation of its terminal hydroxyl group (demonstrated with green arrows). All pathways converge in ceramides.

Sphingolipids have a structural feature of a sphingosine backbone that is comprised of an alkyl chain of 18 carbon atoms with one to three hydroxyl groups and one amino group (**Figure 1A**). To the amino group, different functional groups can bind to yield e.g., sphingosine-1-phosphate (S1P), SM, and ceramide (11). The most abundant sphingolipid in majority of mammalian cells, representing 85% of all sphingolipids, is SM (12). In SM, the sphingosine backbone is bound to a fatty acid tail via the amino group and to a phosphocholine group via the terminal hydroxyl group (**Figure 1A**). SM is produced in the luminal leaflet of *trans-*Golgi lumen membranes from ceramide, which is provided by the endoplasmic reticulum (ER). SM is transported to the plasma membrane by vesicular traffic, where it accumulates in the exoplasmic leaflet (13). Except for maintaining plasma membrane structure, SM is enriched in the endocytic recycling compartment and the *trans*-Golgi network, and can control the actions of growth factor receptors and matrix proteins as well as serve as a binding site for different micro-organisms (14). SM is also a binding partner for cholesterol, influencing cholesterol homeostasis and forming a SM/sterol concentration gradient along the secretory pathways (15). In addition, SM may be a critical source of phosphocholine needed for mineralization (4, 10). Several investigations have demonstrated that abnormal SM metabolism results in abnormalities in the mineralization of the bone matrix (4, 16, 17).

## Sphingomyelin metabolism

The metabolism of SM is highly regulated and involves multiple bioactive sphingolipids. SM can be synthesized *de novo*, through ceramide, from precursors such as palmitoyl CoA and serine (**Figure 1B**, blue pathway) (12). Production of ceramide takes place on the cytosolic surface of the ER (13). Ceramide is then transported to the Golgi complex, where it serves as a substrate for sphingomyelin synthase (SMS) and other sphingolipid-generating enzymes (10). Both vesicular and nonvesicular transport mechanisms can mediate ceramide transport (13).

SMS is a membrane-bound enzyme that has two isoforms, SMS1 and SMS2. SMS2 is found in the plasma membrane and in the Golgi apparatus, whereas SMS1 is localized only in the Golgi (18). SMS catalyzes the transfer of phosphocholine, cleaved from phosphatidylcholine (PC), onto ceramide, generating SM and diacylglycerol (DAG) (**Figure 1B**, red pathway) (12). SM is then delivered by vesicular transport to the plasma membrane (19). SMS2 is also able to catalyze the reverse reaction. However, SMS2 can only regenerate ceramide but is unable to release phosphocholine, instead a histidine-phosphocholine intermediate is formed. Regeneration of phosphocholine from sphingomyelin is done by sphingomyelinases (SMases). SMases fall into three categories depending on their pH optima: acidic, alkaline, and neutral (10, 12). Sphingomyelin phosphodiesterase 3 (SMPD3), one of the four neutral SMases, is largely confined to the bone, cartilage, and brain tissue (17). Ceramide can also be converted to sphingosine-1-phosphate (S1P) through hydrolysis of its fatty acid residue and subsequent phosphorylation of its terminal hydroxyl group (**Figure 1B**, green pathway) (10). For lipids exiting the sphingolipid pool, only a single irreversible catalytic pathway exists: Sphingosine-1-phosphate lyase (S1P lyase) breaks the sphingosine backbone of S1P generating non-sphingolipids (20).

The generation and degradation of SM and the related bioactive lipids are intertwined. Hence, the regulation of these lipids could be affected by the enzymes involved in the metabolism of SM. Due to the interconnectedness of these lipids, changes in one causes a “ripple” effect in the others as a new equilibrium between the substrates sets. Furthermore, there are great variations in these lipids’ concentrations. Since SM has a tenfold higher concentration than ceramide, for instance, even minimal changes in SM can have a large impact on ceramide levels (21).

SM is the preferred binding partner of cholesterol. SM produced in the lumen of the *trans*-Golgi and at the outer leaflet of the plasma membrane provides a thermodynamic trap for cholesterol synthesized in the ER, contributing to the formation of a SM/sterol gradient along the secretory pathway (15). ER and *cis*-Golgi membranes are characterized by low sphingolipid and sterol content while the plasma membrane and *trans*-Golgi have a high sphingolipid and sterol content (22). This nonrandom SM gradient is important for maintaining ER- and plasma membrane specific lipid composition and fundamental for physical membrane properties that help specify organelle identity and function.

## Osteoporosis

Recent research has identified a wide range of illnesses affecting skeletal homeostasis, and often with a genetic basis. Monogenic disorders are caused by a single-gene mutation, which is usually germline but occasionally somatic, while oligogenic or polygenic conditions involve multiple genetic variants (23). Osteoporosis is most commonly polygenic and related to aging, or secondary to other illnesses. However, primary osteoporosis may present already in childhood and is then usually a monogenic disease (24). *SGMS2*-related osteoporosis belongs to this group of monogenic metabolic bone disorders (25).

## SGMS2-related osteoporosis

The rare autosomal dominant inherited bone disease named calvarial doughnut lesions with bone fragility (CDL) with or without spondylometaphyseal dysplasia (OMIM #126550) was described more than 50 years ago (26, 27). However, its genetic cause – mutation in *SGMS2* – was identified only in 2019 (4). In humans, *SGMS2* is located on chromosome 4 and codes for a 365 amino acid protein – sphingomyelin synthase 2 (SMS2). So far, three heterozygous variants have been detected by next-generation sequencing and confirmed by Sanger sequencing in 32 affected subjects from 12 unrelated families (4–8). The reported variants include a c.148C>T variant, which introduces a premature stop codon in exon 2 (p.Arg50*) and yields a truncated enzyme, and two missense variants, c.185T>G (p.Ile62Ser) and c.191T>G (p.Met64Arg) (4, 5). The study by Pekkinen et al. (4) was the first to link aberrant SM metabolism to a bone disease, highlighting the importance of sphingolipids for bone growth and development.

The clinical presentations of *SGMS2*-related osteoporosis range from childhood-onset osteoporosis with low BMD and skeletal fragility with or without sclerotic doughnut-shaped lesions in the skull to a severe spondylometaphyseal dysplasia with neonatal fractures, long-bone deformities, and short stature (4–8, 26). Additionally, glaucoma was diagnosed in two individuals from one affected family harboring a p.Arg50* mutation, as described by Pekkinen et al. (4). Interestingly, the association of glaucoma with *SGMS2* was further strengthened when Collantes and coworkers described a Filipino family harboring the *SGMS2* p.Arg50* mutation, with characteristic skull lesions and juvenile onset open angle glaucoma (7).

Thus far, several single patients as well as larger multigenerational families with heterozygous *SGMS2* variants have been reported. Mutations p.Ile62Ser and p.Met64Arg, that give rise to a more severe phenotype with neonatal fractures, severe short stature, and spondylometaphyseal dysplasia, have been reported in 3 affected subjects in 2 families (4). The p.Arg50* variant, associated with a milder phenotype, is more common and has been described in 29 subjects in 10 families. A more detailed description of the clinical data of the patients with a *SGMS2* p.Arg50* mutation is summarized in **Tables 1A**, **1B**, and presented separately for each family. In addition to the skeletal phenotype, patients portray various neurological manifestations, which will be covered in more detail later in the text. It remains partly unclear how the *SGMS2* variants lead to skeletal fragility and what explains the significant phenotypic differences between patients with the more common p.Arg50* variant and those with the missense variants.

Table 1Clinical findings in 29 subjects with a SGMS2 p. Arg50* variant.

**Table:** 

A.	Pekkinen et al. (4)									
**Family**	**Family 1**			**Family 2**	**Family 3**					**Family 4**
**Relationship**	**Index**	**Father**	**Father´s mother**	**Index**	**Index**	**Sister**	**Brother**	**Mother**	**Mother´s mother**	**Index**
Path. variant	p.Arg50*			p.Arg50*	p.Arg50*					p.Arg50*
Sex	Female	Male	Female	Male	Female	Female	Male	Female	Female	Female
Age (yrs)	22	59	85 (dec)	27	9	12	4	37	60	6
Ethnicity	Finnish			Finnish	Caucasian (USA)					N. European
Height (SD)	+0.4	−0.1	N/A	−1.1	−2.7	−2	−3.2	−1.6	−0.2	−0.8
Peripheral fx	6	>9	>14	9	0	1	0	2	15	6
Spinal fx	Yes	Yes	Yes	Yes	Yes	Yes	Yes	Yes	No	Yes
BMD Z score	−1.4 (at 12 yrs, BT)	−1.9 (at 48 yrs, BT)	N/A	−3.4 (at 15 yrs, BT)	−5.3 (at 5 yrs)	−5.2 (at 8 yrs)	−5.8 (at 4 yrs)	−3.8 (at 34 yrs)	N/A	−15.5 (at 5 yrs, BT)
Skeletal dysplasia	Mild scoliosis	Mild scoliosis	Mild scoliosis	Mild scoliosis	Mild scoliosis	None	None	None	None	None
Skull findings	Few sclerotic lesions	Multiple sclerotic lesions	Irregular diffuse thickening	One sclerotic lesion	Normal	Few sclerotic lesions	Normal	Normal	Sclerotic lesions	Normal
Feature of ocular and auditory systems	Congenital glaucoma	Oculomotorius, and trochlearis paresis	Glaucoma, oculomotorius, and abducens paresis	None	None	None	None	None	None	Mild myopia
Others	Pain and swelling in knee and ankle joints	Chronic duodenal inflammation, abdominal pain, sleep apnea	Diverticulosis, peptic ulcers, atherosclerosis, asthma, hypertension, chronic atrial fibrillation	Mild facial dysmorphia; low nasal bridge, midfacial hypoplasia, colitis	Upper thoracic syringohydro-myelia	None	None	None	Constipation, bone pain (arms), joint pain (ankles and knees)	None

**Table:** 

B.	Robinson et al. (5)			Basalom et al. (6)				Collantes et al. (7)	Whyte et al. (8)			
**Family**	**Family 1**	**Family 2**		**Family 1**			**Family 2**	**Family 1**	**Family 1**			
**Relationship**	**Index**	**Index**	**Father**	**Index**	**Son**	**Mother**	**Index**	**6 family members**	**Index**	**Mother**	**Mother´s mother**	**3 family members**
Path. variant	p.Arg50*	p.Arg50*		p.Arg50*			p.Arg50*	p.Arg50*	p.Arg50*			
Sex	Female	Male	Male	Female	Male	Female	Male	N/A	Male	Female	Female	N/A
Age (yrs)	22	12	40	29	3 months	63	16	Mean 25.5	6	30	59	N/A
Ethnicity	French-Canadian ancestry	French, Swedish, English, French-Canadian descent		French-Canadian ancestry			N/A	Filipino	American kindred of Scandinavian heritage			
Height (SD)	−1.4	+0.8	N/A	-1	N/A	−2.3	−1.5	N/A	N/A			
Peripheral fx	1	3	0	~ 40	0	19	>4	N/A	Multiple	Yes	N/A	N/A
Spinal fx	Yes	Yes	No	No	No	No	Yes	N/A	Multiple	Yes	N/A	N/A
BMD Z score	−4 (at 5 yrs, BT)	−3.1 (at 8.7 yrs, BT)	N/A	−3 (at 8 yrs, BT)	−1	N/A	−2 (at 16 yrs, BT)	N/A	Low spinal BMD			
Skeletal dysplasia	Mild scoliosis, mild genu valgum secondary to tibia bowing	Mild scoliosis, mild genu valgum secondary to tibia bowing	None	"bone in bone” appearance of the vertebral bodies	Osteopenia with linear osteo-condensation of the tibial proximal epiphyses	Osteo-porosis	Juvenile osteo-porosis	N/A	Occipital protrusion and increased cranial digital markings	N/A	Thinned outer cortex with sudden transition to large fibrous areas	N/A
Skull findings	N/A	Cobble stone appearance	N/A	None	None	Doughnut lesions	Doughnut lesions	Skull abnormalities	Ill-defined lytic area with palpable depression appeared in anterior skull	N/A	Calvarial lesions	N/A
Feature of ocular and auditory systems	N/A	Myopia	Myopia	N/A	N/A	Unilateral ocular palsies	N/A	83% blind in at least one eye, glaucomatous optic nerves	N/A	N/A	Hearing loss	N/A
Others	Obese	Delayed loss of primary teeth, obese	Dental crowding	None	None	None	None	Juvenile onset open angle glaucoma	N/A	N/A	N/A	N/A

Path. Variant, pathogenic variant; yrs, year; dec, deceased; N. European, Northern European; N/A, not available; fx, fracture; BF, before treatment. Only the top row should be in gray. The rows that start with family and relationship should be similar as the rest of the rows underneath. Meaning that these rows should be in white and "family" and "relationship" should be in bold while the rest of the text in these rows should not be bold.

## Bone tissue characteristics and expression of SGMS2 in tissues and cells

Transiliac and femoral bone biopsy samples from patients with *SGMS2*-related osteoporosis reveal reduced mineral content and decreased bone volume with unorganized collagenous network (4, 5, 8). Mäkitie et al. have demonstrated that patients harboring a p.Arg50* mutation have a discorded collagenous apposition, their osteocyte lacunae appear too large and the lacuna-canalicular network is extremely distorted and short spanned (28). In human tissues, *SGMS2* transcripts have been detected in brain, heart, kidney, liver, muscle, and stomach (29). *SGMS2* expression has also been detected in primary chondrocytes isolated from patients with osteoarthritis (30). In mice, *sgms2* is highly expressed in cortical bone, vertebrae, kidney, and liver (4). *In vitro* studies performed by Pekkinen et al. showed that cultured murine osteoblasts, bone marrow macrophages and osteoclasts expressed *sgms2* at similar levels (4). Results on patients’ bone biopsies in the Pekkinen et al. study also suggested that osteoclast numbers may be increased based on bone resorption parameters. However, osteoclast formation and function *in vitro* were normal, as analyzed from peripheral blood monocytes from 2 patients with a p.Arg50*mutation (4).

## Enzymatic activity and cellular location of the SMS2 variants

**Figure 2:**
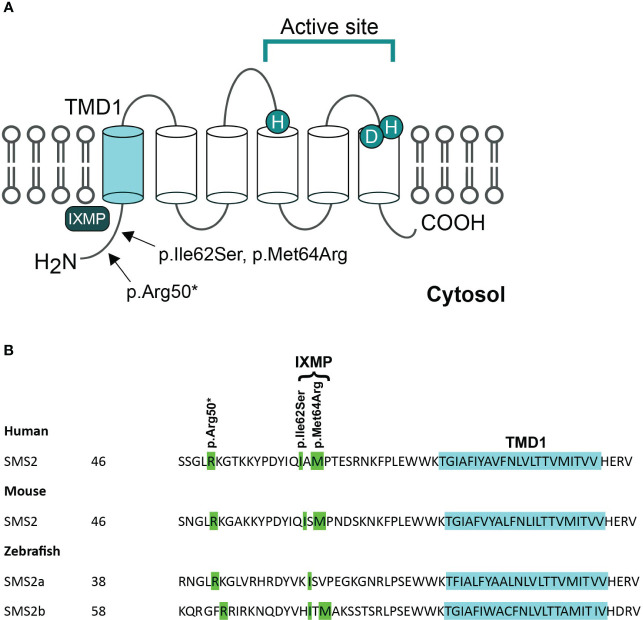
Pathogenic variants of SMS2. **(A)** Predicted membrane topology of SMS2 indicating active site residues and positions of 3 residues substituted in pathogenic SMS2 variants (p.Arg50*, Ile62Ser, Met64Arg). **(B)** SMS2 sequence alignment of the region immediately upstream of transmembrane domain 1 (TMD1) in human, mouse, and zebrafish. Pathogenic SMS2 variants and the ER export defected by Ile62Ser- and Met64Arg-variations are indicated. Database accession numbers for the sequences are human SMS2, Q8NHU3; mouse SMS2, Q9D4B1; zebrafish SMS2a, B8A5Q0; zebrafish SMS2b, Q6DEI3. Adapted and reprinted by permission from JCI Insight (Creative Commons Attribution 4.0 International License (CC BY 4.0)) (Pekkinen et al., (4), copyright 2019).

SMS2 is a multi-membrane spanning protein that primarily contributes to sphingomyelin synthesis and homeostasis at the plasma membrane. The three pathogenic variants of *SGMS2* (p.Arg50*, p.Ile62Ser and p.Met64Arg) are all located in the N-terminal part of the protein in the region immediately upstream of transmembrane domain 1 (TMD1) (**Figure 2**) (4). Variants p.Ile62Ser and p.Met64Arg do not have an effect on SMS2 enzymatic activity. Instead, due to the missense variants, SMS2 is unable to exit the ER because their N-terminal cytosolic tails lack a functioning independent ER export signal (31). Sokoya et al. demonstrated that isoleucine at position 62 and methionine at position 64 in SMS2 are part of a conserved sequence motif, IXMP, which is located 13–14 residues upstream of the first membrane span and is part of this ER export signal (**Figure 2**). By transfecting SMS2^I62S^ and SMS2^M64R^ constructs into Hela cells, they detected the subcellular location of the SMS2 variants with immunofluorescence microscopy, and revealed that SMS2^I62S^ and SMS2^M64R^ were both retained in the ER, while wild type SMS2 localized to the Golgi and the plasma membrane (31). The SMS2 p.Arg50* variant is predicted to result in a truncated enzyme lacking the entire transmembrane helices including the active sites of the enzyme (**Figure 2**) (4). However, Sokoya and co-workers have hypothesized that the nonsense p.Arg50* variant produces a shortened yet functional enzyme with methionine at position 64 serving as an alternative translation initiation site (31). In addition, they hypothesized that the p.Arg50* variant is exported out of ER but fails to reach the plasma membrane and mislocalizes to the *cis*/medial Golgi (31).

The SMS2 missense variants also enhance *de novo* SM biosynthesis, based on elevated triacylglycerol levels in *SGMS2-*mutated patient-derived fibroblasts (4). The elevated triacylglycerol levels are likely to result from rapid conversion of the SM synthesis byproduct, DAG, to triacylglycerol. Therefore, it is anticipated that the onset of the disease is a result of improperly targeted bulk SM production rather than a decreased ability to synthesize SM (4, 31). This finding implies that pathogenic SMS2 variants accumulate SM in the ER and display a disrupted SM asymmetry at the plasma membrane due to altered subcellular organization of SM and cholesterol. In addition, Sokoya and co-workers discovered that pathogenic SMS2 variants significantly alter the ER glycerophospholipid profile (31). These changes include an increased degree of phospholipid desaturation and an increase in cone-shaped ethanolamine-containing phospholipids, which may be a cellular adaptation to the SM-mediated rigidification of the ER bilayer (31). Pathogenic SMS2 mutations may therefore severely impair the ability of cells to maintain nonrandom lipid distributions in the secretory pathway, which may be essential for osteogenic cells’ ability to form bone (31). However, it remains unknown exactly how the pathogenic SMS2 variants affect the subcellular organization of SM and how cholesterol contributes to the development of osteoporosis and CDL in affected patients.

## Potential impact of disrupted SM gradients on bone formation

**Figure 3:**
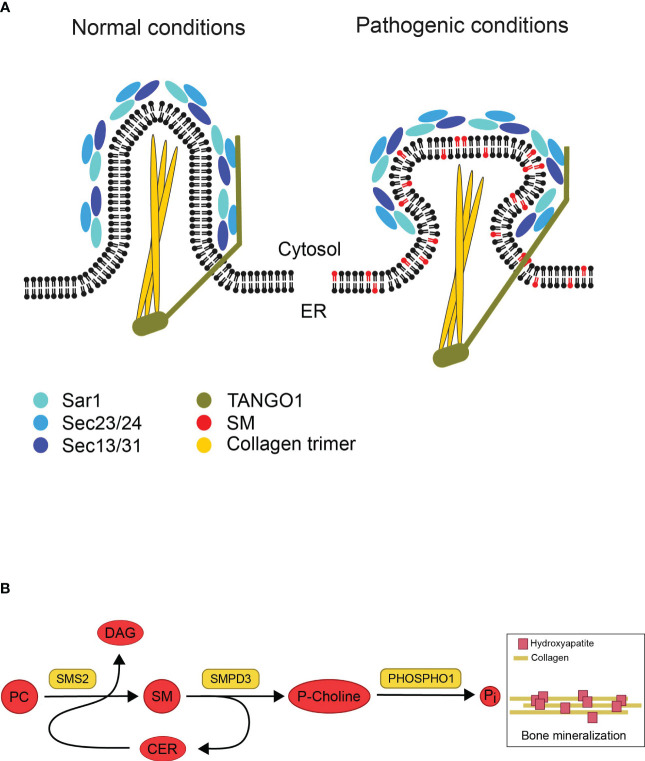
Illustrations based on potential impact of disrupted SM gradients on bone formation. **(A)** Model showing potential effects of pathogenic SMS2 variants on collagen secretion during bone formation. Collagen synthesis starts in the ER. Under normal conditions, the COPII coat proteins (Sar1, Sec23/24, Sec13/31) and the accessory protein TANGO1 are assembled as pro-collagen trimers leave the ER in secretory vesicles. The rigidifying impact of SM on both ER bilayer leaflets would hinder the development of these big cargos (pathogenic conditions). This, in turn, would stop collagen trimers from being properly exported from the ER, which would negatively impact bone formation. Adapted and reprinted from Gillon et al. (33) (Biochim Biophys Acta; 1821(8): 1040–1049). **(B)** Model describing how sphingomyelin metabolic enzymes contribute to the mineralization of bone. In the exoplasmic leaflet of the plasma membrane, sphingomyelin (SM) is broken down by the enzyme sphingomyelin phosphodiesterase 3 (SMPD3) to produce ceramide (Cer) and phosphocholine (P-Choline). Phosphatase (PHOSPHO1) uses P-Choline as a substrate to release phosphate and promote bone mineralization. The plasma membrane-resident sphingomyelin synthase SMS2 produces diacylglycerol (DAG) as a byproduct while regenerating SM from Cer released by SMPD3 utilizing phosphatidylcholine (PC) as a head group donor. The osteoblast surface SMPD3 and SMS2 enzymatic activities work together to provide the constant flow of phosphocholine needed for normal bone mineralization. Adapted and reprinted by permission from JCI Insight (Creative Commons Attribution 4.0 International (CC BY 4.0)) (Pekkinen et al., (4), copyright 2019).

Based on the Pekkinen et al. study, *SGMS2* transcript levels are highest in cortical bone and vertebrae in murine model (4), indicating that the effect of pathogenic variants on the lipid composition of secretory organelles could be severe in bone cells. Bone is formed when collagen fibrils are deposits into a matrix that will mineralize, in the presence of Ca^2+^ and inorganic phosphate (P_i_), when hydroxyapatite crystals grow within and between the newly synthesized collagen fibrils (32). Collagen synthesis begins in the ER as pre-collagen, which leaves the ER as pro-collagen through coat protein complex type II (COPII) vesicles. Pro-collagen’s ability to leave the ER is dependent on the COPII coat, which is made up of the essential elements Sar1, Sec23/24, and Sec13/31. In addition, TANGO1, an ER-resident transmembrane protein, is required for packaging pro-collagen fibers into COPII vesicles (**Figure 3A**) (34). Mutations in COPII components and TANGO1 have been reported to selectively disrupt procollagen export from the ER and cause insufficient bone mineralization (35–37). One scenario is that *SGMS2* variants could impair the formation of secretory vesicles containing pro-collagen due to the rigidifying effect of SM on both leaflets of the ER bilayer (**Figure 3A**). This would prevent proper export of collagen from the ER and impact bone formation.

Another possible explanation is that bone mineralization is adversely affected by pathogenic SMS2 variant due to disturbed SM asymmetry at the plasma membrane in osteogenic cells. When bone mineralizes, matrix vesicles bud off from osteoblasts’ apical membrane and deposit their phosphate- and Ca^2+^-rich contents at the mineralization site (32). Both SMS2 and SMases can break down SM into ceramide in the cytosolic leaflet of the plasma membrane, but unlike SMases, SMS2 is unable to release phosphocholine (12). Phosphocholine can be utilized to produce P_i_ for the mineralization process, where P_i_ precipitates into hydroxyapatite when combined with Ca^2+^ (**Figure 3B**) (10). During cartilage mineralization, matrix vesicles, the initial sites of mineral production, have been seen to degrade SM rapidly. SMases like SMPD3, which are present in matrix vesicles, are likely responsible for the reduction of SM. Due to this degradation, phosphocholine (P-Choline) is produced, which PHOSPHO1 can employ to liberate phosphate (10, 38, 39). Even though SMS2 itself cannot release phosphocholine, it may regenerate SM from ceramide released by SMPD3, and refill the SM pool used by SMPD3 to set free phosphocholine during bone mineralization. The regeneration of SM by SMS activity would guarantee a steady supply of phosphate (4). This concept could explain how pathogenic SMS2 variants may cause a premature exhaustion of the lipid-based phosphate store thus interfering with normal bone mineralization.

## Sphingomyelin synthase and SM metabolism in the skeletal system

In addition to the study by Pekkinen et al. (4), several other studies have demonstrated sphingomyelin synthases’ various key roles in bone homeostasis. Matsumoto and collaborators, who examined the role of sphingomyelin synthases in the skeletal development of mice, discovered that whereas SMS2 deficit did not affect bone formation, SMS1 deficiency did (16). Compared to control mice, SMS1-deficient mice had decreased cortical and trabecular bone mass, lower BMD, and delayed mineral deposition. The osteoid volume and the osteoid development in these animals increased dramatically, a further evidence for impaired mineralization. Also, when stimulated with bone morphogenic protein 2 (BMP2), tamoxifen-inducible SMS1-deficient calvarial osteoblasts demonstrated a significant decrease in the expression of several bone structural components and reduced mineralization (16).

Even though the nature of SMS2’s effect on bone is uncertain, it has been suggested that SMS2 indirectly affects osteoclast differentiation through osteoblasts (40). Osteoclasts are of hematopoietic lineage origin and their synthesis is being controlled by macrophage colony-stimulating factor (M-CSF) and nuclear factor b ligand (RANKL). RANKL, secreted by osteoblasts, regulates factors that control bone resorption, including parathyroid hormone (PTH), 1,25(OH)_2_ vitamin D, and interleukins 6 and 11 (IL-6, IL-11) (41). Yoshikawa and collaborators showed that deletion of SMS2 with siRNA affected osteoclastogenesis through the 1,25(OH)_2_D pathway. 1,25(OH)_2_D binds to vitamin D receptor (VDR) which then dimerizes with retinoid-X-receptor-α (RXRα) to regulate gene expression, including RANKL. RXRα is a receptor that exerts its action by binding, as homo- or heterodimers, to specific sequences in the promoters of target genes and regulating their transcription (42). A siRNA-mediated SMS2 knockdown in mouse primary osteoblasts reduced the expression of RXRα mRNA and, as expected, the expression of RANKL after 1,25(OH)_2_D stimulation. In consequence, the number of differentiated osteoclasts was significantly reduced (40). To date, the mechanism behind RXR downregulation in these cells is still unknown. Interestingly, the suppression of osteoclastogenesis would increase bone mass (43), not decrease it as SMS-deficiency studies show (4, 16). As earlier described, bone biopsies from the patients harboring a *SGMS2-*variant in the Pekkinen et al. study also suggest that osteoclast numbers might be increased based on bone resorption parameters (4). However, the *in vitro* study on peripheral blood monocytes showed normal osteoclast formation and function (4). These findings imply that alterations in the mineralization process itself rather than an abnormally high bone turnover might cause skeletal abnormalities in SMS-deficient conditions.

Abnormal activity of other sphingomyelin metabolizing enzymes has been linked to bone abnormalities in mice. Sphingomyelin phosphodiesterase 3 (SMPD3) has been recognized as an essential regulator of development in skeletal and cartilaginous tissues (10). Deletion of Smpd3 in mice leads to severe skeletal abnormalities, poor mineralization of bone and cartilage, and features consistent with severe osteogenesis imperfecta (44, 45). S1P has bone-specific roles, specifically in osteoblasts and osteoclasts where S1P acts as an osteoclast-osteoblast coupling factor to coordinate bone resorption and bone formation (46). According to previous studies, the S1P balance in bone is essential for maintaining skeletal homeostasis since disturbance of this balance in mice results in osteopetrosis and osteoporosis (47). Moreover, DAG, the byproduct of sphingomyelin synthase, activates protein kinase C (PKC) in cells (48). It has been shown that PKCδ, a novel isoform of PKC, is essential for the signaling of Wnt3a-induced osteoblastogenesis since PKC mutant mice exhibit inadequate embryonic skeletal development (49). The PKC pathway is also used by Wnt7b to stimulate osteoblast differentiation (49). However, the DAG itself has not been implicated in bone formation.

## Neurological findings in SGMS2-related osteoporosis

In addition to the bone phenotype, several patients with a pathogenic *SGMS2* variant exhibit neurological symptoms (**Table 2**) (4–6, 8). The most prevalent findings were isolated cranial nerve palsies that are transient, recurrent, and spontaneously remitting. Most commonly, these have been peripheral facial nerve palsies but oculomotor, trochlear and abducens nerves have also been affected. Diagnostics of clinical evaluations were repeatedly normal, showing no indication as to what these palsies could be attributed to. Other reported neurological findings included migraines, depression, dystonia, trigeminal neuralgia, sensory neuropathy, ataxia and absent or decreased reflexes (4–6, 8).

**Table 2:** Nerological features in 11 subjects with a pathogenic *SGMS2* variant.

	Family	Relationship	Pathogenic variant	Sex	Age (y)	Neurological features
Pekkinen et al. (4)	Family 1	Index	p.Arg50*	Female	22	Migraine, transient facial nerve palsies, right hand dystonic tremor
		Father		Male	59	Facial nerve palsies, oculomotorius, and trochlearis paresis, canalis carpi, trigeminus neuralgia, cephal-algia, clonic Achilles reflex, depression
		Father´s mother		Female	85 (deceased)	Alzheimer’s disease, transient brain ischemic attack, subdural hematomas, transient facial nerve palsies, oculomotorius, and abducens paresis, depression
	Family 3	Mother´s mother	p.Arg50*	Female	60	Migraine, headaches, transient facial nerve palsies
	Family 5	Index	p.Ile62Ser	Female	43	Facial paresis, diplopia, sensory neuropathy, ataxia, limited patellar, Achilles, and upper extremity reflexes
		Son		Male	7	Unilateral facial nerve palsies
	Family 6	Index	p.Met64Arg	Male	11	Facial diplegia, decreased bulbar function, hypotonia, mild delay in motor development
Robinson et al. (5)	Family 1	Index	p.Arg50*	Female	22	Migraines with aura, normal neurological examination
	Family 2	Index	p.Arg50*	Male	12	Episodes of unresponsiveness, bowel incontinence
Basalom et al. (6)	Family 1	Mother	p.Arg50*	Female	63	Unilateral ocular palsies
Whyte et al. (8)	Family 1	Index	p.Arg50*	Male	6	Transient facial nerve palsies

## Sphingomyelin metabolism in neurological diseases

In the mammalian body, the nervous system is one of the tissues with the highest lipid complexity and content. The formation and preservation of the functional integrity of the central nervous system (CNS) depends on sphingolipids, which are particularly abundant in the brain (50). Alterations in neural membrane glycerophospho- and sphingolipid composition can influence crucial intra- and intercellular signaling and alter the membrane’s properties (51). In the CNS, SM is enriched in oligodendrocytes and myelin, and plenty of evidence indicates that SM metabolism plays an important role in neurodegenerative and psychiatric diseases (50).

Cerebral ischemia, a condition where the brain is deprived of its blood supply and causes neurodegeneration, has been linked to SM metabolism (12). After brain ischemia, SM hydrolysis and consequent pro-apoptotic ceramide production take place (52, 53). Functional studies also demonstrate that inhibition of neutral SMases suppresses the ischemia-related apoptotic process both *in vitro* and *in vivo* (54).

The role of lipids in Alzheimer’s disease, characterized by deposition of amyloid-beta (Aβ) plaques, has been studied extensively and data suggests that lipid composition of the brain may be involved in neurodegenerative processes (55). Deregulation of SM and ceramide, in particular, have been associated with the disease (12) and may promote abnormal amyloid processing (56). Early pathogenesis of Alzheimer´s disease has been suggested to associate with SM located in the cerebrospinal fluid (CSF), since CSF SM is positively correlated with Aβ and tau levels in high-risk healthy patients and in patients in the prodromal stage of Alzheimer’s disease (57, 58). Decreased SM levels and increased ceramide levels, together with an elevated expression of acid SMase, have also been consistently observed in Alzheimer’s disease brains (59, 60).

Clinical hallmarks of Parkinson´s disease are Lewy bodies or α-synuclein (61). Lysosomal pathways have been found to degrade α-synuclein, leading to the hypothesis that their impairment may play a role in the development of Parkinson disease (62). Two variants of the lysosomal acid SMase have been reported to increase the risk of Parkinson disease significantly, suggesting that a disturbed SM metabolism may play a role (63, 64).

## Model organisms with SGMS2 variations

Since the exact mechanism by which *SGMS2* variants alter SM metabolism in bone remains unclear, genetically modified animal models are a good method to mimic the disease and characterize skeletal pathology. Sms2 knockout (KO) mice are available (65, 66). Hailemariam and coworkers developed Sms2 KO mice already in 2008 and 3 years later, in 2011, Mitsutake and coworkers followed their lead (65, 66). These Sms2 KO mice have been utilized in multiple studies during the last decade (**Table 3**). Mitsutake and coworkers showed that by removing Sms2 in mice, diet-induced obesity and insulin resistance are reduced (65). Hailemariam and coworkers revealed that *sgms2* KO mice had a diminished NFκB response to inflammatory/immunologic stimuli (66). However, neither study reported any obvious bone abnormalities (65, 66). Despite this, a bone phenotype may have gone unnoticed. On the other hand, it is possible that removal of SMS2 is not sufficient to cause a bone phenotype, since SMS1 also produces SM. Instead, it may be necessary to induce pathogenic variants of *sgms2*, to provoke mislocalization of Sms2 in the cells, to be able to engender bone abnormalities. Such a study has not yet been reported. No neurological manifestations in *sgms2* KO mice have been described (65, 66).

**Table 3:** SMS2 modified animal models in various mouse studies.

Reference	Animal model	Study	Generated
Honma et al. (67)	SMS2 knock out (KO) mice	Skin study	Generated by homologous recombination using targeted vectors
Chiang et al. (68)	Hepatocyte-specific Sms1 KO/global Sms2/global Smsr triple KO mice	Liver study	Hepatocyte-specific Sms1/global Sms2 double KO mice (Li et al. (69)) and global Smsr KO mice (Ding et al. (70))
Ou et al. (71)	SMS2 KO and 3H9/Sgms2 KO mice	Lupus erythematosus study	SMS2 KO (Liu et al. (72)) 3H9 knockin mice (Chen et al. (73))
Chiang et al. (74)	Hepatocyte-specific Sms1 KO/global Sms2/global Smsr triple KO mice	Liver study	Hepatocyte-specific Sms1/global Sms2 double KO mice (Li et al. (69)) and global Smsr KO mice (Ding et al. (70))
Sakai et al. (75)	SMS2 KO mice	Epidermis study	Mitsutake et al. (65)
Li et al. (69)	Hepatocyte-specific Sms1/global Sms2 double KO mice	Liver study	Sms2 KO mice (Liu et al. (72))
Sugimoto et al. (76)	SMS2 KO mice	Liver study	Mitsutake et al. (65)
Deng et al. (77)	SMS2 KO mice	Cancer study	Liu et al. (72)
Taniquchi et al. (78)	SMS2 KO + SMS2 KO mice, which have a floxed allele for SMS1 (SMS2-/-;SMS1f/f)	Cancer study	SMS2 KO (Mitsutake et al. (65)) and SMS2−/− mice with a SMS1 fl/fl (Ohnishi et al. (79))
Matsumoto et al. (16)	Sp7-Cre;SMS1f/f;SMS2−/− and ERT2-Cre;SMS1 f/f;SMS2−/− mice generated from SMS2−/−;SMS1f/f mice	Analyzed the phenotype of a conditional knockout mouse; Sp7-Cre;SMS1f/f;SMS2-/- mouse	SMS2−/− mice with a SMS1 fl/fl (Ohnishi et al. (79))
Xue et al. (80)	SMS2 KO mice	Cerebral ischemia study	Hailemariam et al. (66)
Gupta et al. (81)	SMS2 KO mice	Pulmonary function study	Liu et al. (72)
Nomoto et al. (82)	SMS2 KO mice	Skin study	Mitsutake et al. (65)
Ohnishi et al. (79)	SMS2-/-;SMS1f/f generated from SMS2 KO mice	Dextran sodium sulfate (DSS)–induced murine colitis study	SMS2 KO mice (Mitsutake et al. (65))
Wang et al. (83)	SMS2 KO mice	Learning ability study	Mitsutake et al. (65)
Sakamoto et al. (84)	SMS2 KO mice	Study on SMS2 function and properties	Mitsutake et al. (65)
Sugimoto et al. (85)	SMS2 KO mice + loxP-floxed SMS2 mice	Study on insulin-targeted tissues	SMS2 KO mice (Mitsutake et al. (65))
Sugimoto et al. (86)	SMS2 KO mice	Liver and kidney study	Mitsutake et al. (65)
Wang et al. (87)	SMS2 KO mice	Study on alcohol-induced neuroapoptosis	Liu et al. (72)
Ding et al. (70)	Smsr/Sms2 double KO	Study on SMSr function	SMS2 KO (Liu et al. (72))
Li et al. (88)	SMS2 liver-specific transgenic and SMS2 KO mice	Hepatic steatosis study	Produced in their lab
Lu et al. (89)	SMS2 KO	Study on auditory function	Mitsutake et al. (65)
Subbaiah et al. (90)	SMS2 KO mice	Cholesterol study	Hailemariam et al. (66)
Deng et al. (91)	SMS2 KO mice	Study on neurons	N/A, article not in English
Li et al. (92)	Sms2 KO mice	Insulin study	Hailemariam et al. (66), Liu et al. (72)
Mitsutake et al. (65)	SMS2 KO mice	Study on the physiological function of SMS2	Deletion of the SMS2-exon 2, with a cassette encoding β-galactosidase and a neomycin-selectable marker, homologues recombination
Zhang et al. (93)	SMS2 KO mice	Brain study	Hailemariam et al. (66)
Gowda et al. (94)	SMS2 KO mice	Lung study	Hailemariam et al. (66)
Fan et al. (95)	SMS2/Apoe double KO mice	Atherosclerosis study	Produced in their lab
Qin et al. (96)	SMS2 KO mice	Atherosclerosis study	N/A, article not in English
Liu et al. (72)	SMS2 KO mice	Atherosclerosis study	Hailemariam et al. (66)
Hailemariam et al. (66)	SMS2 KO mice	NFκB activation study	Replaced 90% of SMS2-exon 2, with the neomycin-resistant gene, homologous recombination

Zebrafish is a good vertebrate model to study human skeletal diseases, since zebrafish share similar skeletal elements and ossification types with mammals (97). However, there are several differences between zebrafish and mammals related to bone morphology and function, which need to be taken into account when using zebrafish as a model for skeletal diseases (98). The zebrafish has two orthologues of human *SGMS2*, *sgms2a* and *sgms2b* (99, 100). To date, no *sgms2* KO zebrafish models have been reported. However, our recent work demonstrates that knockdown of *sgms2a*, *sgms2b* and *sgms2a+b* by CRISPR-Cas13d result in defective cartilage and early skeletal element development in comparison to control fish (101). To further elucidate the skeletal abnormalities caused by *sgms2* knockdowns, *sgms2* knockouts and perhaps pathogenic *sgms2* knockins mimicking human pathogenic mutations need to be established. Our study also revealed that *sgms2a, sgms2b, sgms2 a+b* knockdown zebrafish (6 days post fertilization) showed altered locomotor activity and behavioral response to light/dark transition test compared to controls, indicating a possible role of *sgms2* in brain and nervous system function in zebrafish (101).

## Conclusions

Recent human studies indicate that heterozygous variants in *SGMS2* lead to a spectrum of skeletal disorders in which skeletal fragility is the leading manifestation. On the bone tissue level, at least the p.Arg50* variant leads to greatly altered bone architecture and defective mineralization. The molecular and cellular mechanisms behind *SGMS2*-linked osteoporosis are not fully understood, but it is believed that the onset of the disease is a result of improperly targeted bulk SM production rather than a diminished capacity to synthesize SM. The *SGMS2* variants are anticipated to disturb the export of SMS2 from the ER. The missense variants cause the SMS2 protein to be retained in the ER while the p.Arg50* variant is hypothesized to mislocalize the protein to the cis/medial Golgi. This mistargeted SM production results in significant deviations in organellar lipid compositions and membrane properties along the secretory pathway. Different targeted SM production between the missense and the p.Arg50* variants could, therefore, explain the phenotypic differences seen between patients with different *SGMS2* variants.

The relationship between the abnormal subcellular organization of SM and the development of osteoporosis in affected patients remains unknown. One possibility is that pathogenic SMS2 variants affect lipid composition of secretory organelles and prevent proper export of collagen from the ER, affecting bone formation (32). On the other hand, pathogenic SMS2 variants may disturb the SM asymmetry of the plasma membrane in osteogenic cells and negatively affect bone mineralization (31). In cells expressing pathogenic SMS2 variants, it is possible that cytosolically exposed SM is constitutively converted to ceramide prematurely, unintentionally diminishing the fuel that powers matrix vesicle formation. This may hamper a continuous supply of phosphocholine required for normal bone mineralization (4).

The role of SM metabolism in the CNS remains less well characterized. Several CNS disorders, including cerebral ischemia, neurodegenerative diseases, and psychiatric illnesses, have been linked to altered SM metabolism. Extensive clinical evaluation of patients has revealed no apparent cause of patients’ neurological symptoms and they are therefore presumed to be secondary to the altered SMS2 function. However, it is unknown whether these arise directly from changed SM metabolism or if they are merely the outcome of cellular circumstances that are otherwise pathologically changed.

Further research is needed to shed light on the molecular mechanisms leading from genetic variants to bone fragility and whether SM metabolism may provide novel targets for therapeutic intervention. Also, new *sgms2* stable mutant zebrafish lines could be utilized in drug discovery and screening platform. Targeted treatments may also be relevant in other forms of osteoporosis in the general population.

## Author contributions

All authors contributed to the article and approved the submitted version.
